# Synaptic Cell Adhesion Molecules in Alzheimer's Disease

**DOI:** 10.1155/2016/6427537

**Published:** 2016-05-03

**Authors:** Iryna Leshchyns'ka, Vladimir Sytnyk

**Affiliations:** School of Biotechnology and Biomolecular Sciences, The University of New South Wales, Sydney, NSW 2052, Australia

## Abstract

Alzheimer's disease (AD) is a neurodegenerative brain disorder associated with the loss of synapses between neurons in the brain. Synaptic cell adhesion molecules are cell surface glycoproteins which are expressed at the synaptic plasma membranes of neurons. These proteins play key roles in formation and maintenance of synapses and regulation of synaptic plasticity. Genetic studies and biochemical analysis of the human brain tissue, cerebrospinal fluid, and sera from AD patients indicate that levels and function of synaptic cell adhesion molecules are affected in AD. Synaptic cell adhesion molecules interact with A*β*, a peptide accumulating in AD brains, which affects their expression and synaptic localization. Synaptic cell adhesion molecules also regulate the production of A*β* via interaction with the key enzymes involved in A*β* formation. A*β*-dependent changes in synaptic adhesion affect the function and integrity of synapses suggesting that alterations in synaptic adhesion play key roles in the disruption of neuronal networks in AD.

## 1. Synaptic Cell Adhesion Molecules

Cell adhesion molecules (CAMs) are cell surface glycoproteins located at the cell surface plasma membrane of neurons and other cells. CAMs have a large extracellular domain and are either transmembrane proteins or attached to the plasma membrane via a glycosylphosphatidylinositol (GPI) anchor. The extracellular domains of CAMs mediate cell adhesion by either forming homophilic adhesion bonds via binding to the same molecules on cell surface membranes of adjacent cells or interacting heterophilically with other proteins on the cell surface membranes of adjacent cells or in the extracellular matrix [1].

**Figure 1:**
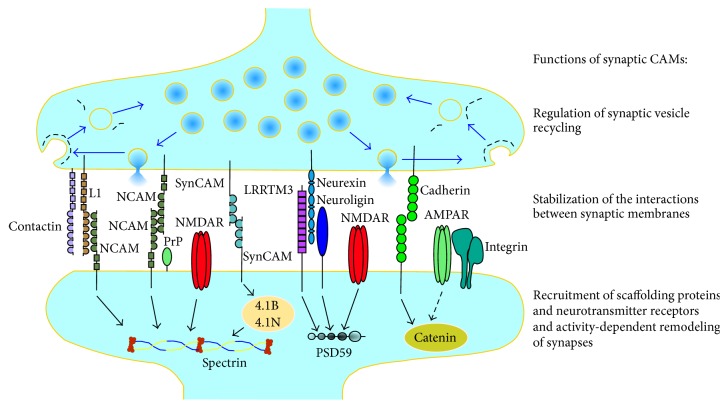
Schematic diagram illustrating examples of synaptic CAMs in glutamatergic synapses. Synaptic CAMs accumulate in synaptic membranes where they form homophilic (e.g., NCAM-NCAM, SynCAM-SynCAM, and cadherin-cadherin) or heterophilic (e.g., L1-NCAM, neuroligin-neurexin, and LRRTM3-neurexin) adhesion bonds, which are important for stabilization of the interactions between synaptic membranes. Presynaptically, CAMs are involved in regulation of synaptic vesicle recycling (blue arrows), which is mediated by coat proteins (black dashed lines) assembled on synaptic membranes to reform synaptic vesicles after exocytosis and neurotransmitter release. Postsynaptically, intracellular domains of synaptic CAMs interact with scaffolding and adaptor proteins (examples of interactions are shown with black arrows), such as spectrin, postsynaptic density protein 95 (PSD95), proteins 4.1B and 4.1N, or catenin, which link synaptic CAMs to postsynaptic glutamate receptors, the *α*-amino-3-hydroxy-5-methyl-4-isoxazolepropionic acid receptors (AMPAR) and N-methyl-D-aspartic acid receptors (NMDAR). Interactions with synaptic CAMs promote the recruitment of scaffolding proteins and neurotransmitter receptors to synapses and are involved in the activity-dependent remodeling of synapses. NCAM: neural cell adhesion molecule, SynCAM: synaptic cell adhesion molecule, PrP: cellular prion protein, and LRRTM3: Leucine-rich-repeat- (LRR-) containing transmembrane protein 3.

CAMs accumulating at synapses between neurons are often called synaptic CAMs and represent members of the major families of cell adhesion molecules, including immunoglobulin superfamily (IgSF) CAMs, cadherins, integrins, neuroligins, and neurexins, and also other cell surface proteins, which mediate cell adhesion, such as cellular prion protein (PrP^c^) and amyloid precursor protein (APP) (Figure 1).

Synaptic CAMs perform numerous functions at synapses (Figure 1). In developing neurons, CAMs promote mechanical stabilization of the contacts between axons and dendrites of neurons [2] and formation of synapses [3]. Synaptic CAMs also play key roles in the establishment of neurotransmission by recruiting other synaptic components, such as synaptic scaffolding proteins, which interact with the intracellular domains of synaptic CAMs, and associated neurotransmitter receptors (Figure 1), and by inducing the maturation of the neurotransmitter release machinery [4]. In mature neurons, CAMs play a role in the stabilization of the synapse ultrastructure [5–7], regulation of the neurotransmitter release [8, 9], and synaptic remodeling and plasticity [10–13]. The multiple roles of synaptic CAMs in regulation of synapse formation and function have been described in a number of recent reviews [14, 15] and are discussed here mostly in the context of Alzheimer's disease.

Alzheimer's disease (AD) is a neurodegenerative brain disorder, which predominantly affects the aging population. One of the earliest signs of AD is the loss of synapses [16]. Synapse loss in AD has been linked at least partly to the toxicity induced by A*β*, a peptide that accumulates in the brains of AD patients [17–19]. Synaptic cell adhesion is directly involved in AD pathogenesis, since APP is a precursor protein of the A*β* peptide and also a synaptic cell adhesion molecule playing a role in regulation of synaptic morphology, synaptic plasticity, and hippocampus-dependent behavior [20]. Functions of APP in synapses and molecular mechanisms of A*β* formation are the subject of a number of recent reviews [21–23] and are not discussed here. In this review, we summarize current data on the changes in the levels and function of other synaptic CAMs in AD brains and their complex interactions with A*β* suggesting that abnormal function in different synaptic CAMs can be an important factor contributing to synapse dysfunction in AD.

## 2. Genetic Association between CAMs and AD

The involvement of CAMs in AD is suggested by genome-wide association studies (GWAS). Significantly altered expression of CAM pathway genes in AD was found in the samples from the cerebellum and temporal cortex of AD-affected individuals and AD-nonaffected controls [24]. Besides APP, among synaptic CAMs found to be associated with the risk of AD, PRNP gene coding for PrP^c^ has been identified as an AD susceptibility gene by systematic meta-analysis of AD genetic association studies [25]. The methionine/valine (M/V) polymorphism at codon 129 within the PRNP gene, which represents a known risk factor for Creutzfeldt-Jakob disease (CJD), has also been reported to be a risk factor for early onset AD [26–28].

Single nucleotide polymorphisms (SNPs) in the neural cell adhesion molecule 2 (NCAM2), a synaptic IgSF CAM highly expressed in hippocampal synapses, have been reported as a risk factor related to the progression of AD in the Japanese population [29]. SNPs in the NCAM2 gene also show association with levels of A*β* in the cerebrospinal fluid in humans, suggesting that NCAM2 is involved in the pathogenic pathway to the senile plaques that concentrate in AD brains [30]. In another large GWAS involving over 16,000 individuals, SNPs in contactin-5, another member of the synaptic IgSF CAMs localizing to the presynaptic membranes [31], were shown to be significantly associated with AD [32]. The junctional adhesion molecule 2 (JAM2) is another member of IgSF potentially linked to AD. SNPs in JAM2 were found to be significantly associated with AD [33]. JAM2 is localized to tight junctions in epithelial and endothelial cells but is also expressed in retinal ganglion cells [34]. The link between JAM2 and AD is also suggested by a study reporting chromosomal 21 region duplication spanning 0.59 Mb and comprising JAM2, APP, and some other genes in a patient with AD [35]. Whether JAM2 functions in the regulation of synapses in neurons is, however, not known. The association with AD was also observed for SNPs in the gene coding for the leucine-rich repeat transmembrane neuronal 3 (LRRTM3) synaptic CAM, which is highly expressed in the hippocampus [36]. Meta-analysis of five GWAS also identified the gene coding for neurexin-3 as a gene playing a role in susceptibility to AD in males [37].

## 3. Changes in the Levels of Synaptic CAMs in AD

Changes in the levels of synaptic CAMs in AD brains have been reported in a number of studies performed over the last 25 years. Reduced levels of the largest NCAM isoform with the longest intracellular domain, NCAM180, but not total NCAM levels have been reported in one of the early studies comparing samples from control and AD frontal cortex by quantitative crossed immunoelectrophoresis [38] suggesting changes in the expression of NCAM in AD. In later studies, analysis of control and AD brain sections by immunohistochemistry with antibodies against NCAM found significantly fewer NCAM positive neurons in the frontal cortex of AD-affected individuals when compared to normal aging individuals [39]. In agreement, the levels of NCAM were shown to be reduced in frontal and temporal cortex from AD patients by ELISA [40]. Interestingly, there was little difference in the levels of NCAM in the occipital cortex and hippocampus of control and AD patients [39, 41]. However, immunohistochemical analysis of the AD hippocampus using antibodies against polysialic acid (PSA), a unique carbohydrate attached predominantly to NCAM, revealed an increase in the immunoreactivity and numbers of PSA-NCAM positive neurons in AD hippocampus and especially in the dentate gyrus indicating changes in the posttranslational processing of NCAM [42]. PSA-NCAM is highly expressed in the developing nervous system, but its expression in the mature nervous system is restricted to brain areas undergoing plastic changes [43], suggesting that an increase in PSA-NCAM in AD is related to extensive neuronal remodeling in AD brains.

Levels of contactin-2, a GPI anchored IgSF CAM also called transient axonal glycoprotein 1 (TAG-1), were shown by Western blot to be reduced in the temporal lobe of AD patients [44]. Contactin-2 is cleaved by *β*-site amyloid precursor protein-cleaving enzyme 1 (BACE1) and its levels in AD brains inversely correlate with BACE1 levels and amyloid plaque density [44] suggesting that an increase in BACE1 activity observed in late-onset AD [45–49] results in increased contactin-2 cleavage. BACE-1 also cleaves other synaptic CAMs, such as IgSF CAM L1 and the close homologue of L1 (CHL1) [50, 51]. The intracellular domain of L1 is also cleaved by *γ*-secretase in human carcinoma cells [52], and *γ*-secretase induced proteolytic cleavage of L1 is increased in a mouse model of AD, which carries human APP with the pathogenic Swedish mutation and the L166P mutated human presenilin-1 [53]. Changes in the activity of BACE-1 and *γ*-secretase may therefore affect the expression of a number of other synaptic CAMs in AD brains.

In addition to IgSF CAMs, levels of PrP^c^ analyzed by Western blot were also found to be decreased in the hippocampus of patients with sporadic AD but not with familial AD [54]. Levels of PrP^c^ are also lower in the temporal cortex samples of AD patients [54, 55]. Levels of N-cadherin are also reduced in the temporal cortex of AD patients [56]. In contrast, Western blot analyses have not revealed significant changes in the levels of contactin-5 in the temporal cortex of AD patients [55] and levels of full length N-cadherin in the superior frontal gyrus of AD patients [57]. Levels of platelet endothelial cell adhesion molecule 1 (PECAM1), an IgSF CAM, were also similar in frontal and temporal cortex of control subjects and moderate to severe AD patients [58]. Therefore, expression of only a subset of synaptic CAMs appears to be affected in AD and only in some brain regions.

**Figure 2:**
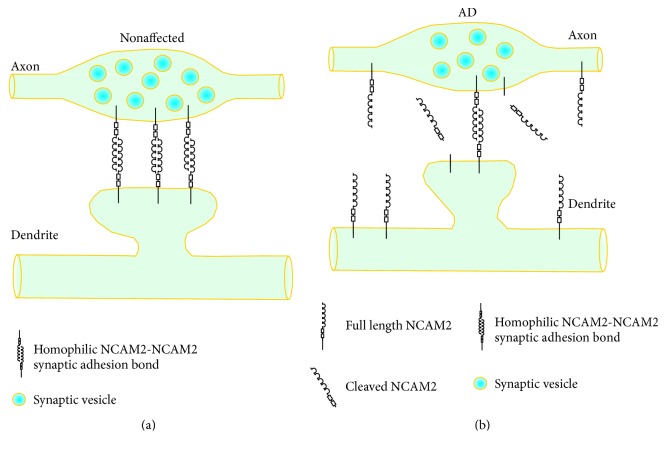
Changes in NCAM2-mediated synaptic adhesion in AD-affected hippocampus. In AD-nonaffected hippocampus (a), NCAM2 accumulates in synapses and plays a role in the synapse maintenance. In AD-affected hippocampal synapses (b), levels of full length NCAM2 are decreased. This decrease is accompanied by an increase in the levels of the proteolytic cleavage products of NCAM2. The overall expression of NCAM2 is also increased probably due to the increase in the levels of extrasynaptic NCAM2.

Interestingly, in a recent study, levels of NCAM2 were shown by Western blot to be increased in the hippocampus of AD patients but strongly reduced in synaptosomes isolated from this brain region [59] (Figure 2). Levels of NCAM2 were not significantly affected in the temporal cortex and cerebellum of AD patients. These observations indicate that changes in the total protein levels or the lack of such changes does not necessarily correlate with the changes in the subcellular localization and function of synaptic CAMs. Changes in the levels of other synaptic CAMs at synapses in AD brains and whether alterations in the overall levels of other synaptic CAMs reflect changes in their synaptic localization remain to be investigated in the future studies.

## 4. Changes in the Levels of the Proteolytic Products of CAMs in AD

In addition to changes in the levels of the full length synaptic CAMs, changes in the levels of the proteolytic products of synaptic CAMs have also been found in AD brains. Interestingly, changes in the proteolytic products of synaptic CAMs do not necessarily correlate with the changes in the total protein levels. While the total levels of N-cadherin appear to be unaffected in the superior frontal gyrus of AD patients, the levels of ectodomain-shed C-terminal fragment of N-cadherin are increased [57]. The levels of the extracellular domains of NCAM2 proteolytically released from the neuronal cell surface are increased in AD hippocampus [59] (Figure 2). This increase in the levels of proteolytic products of NCAM2 inversely correlates with the levels of full length NCAM2 at synapses, while the total levels of NCAM2 are also increased in AD hippocampus [59] (Figure 2). It is therefore possible that changes in the proteolytic products of synaptic CAMs in AD brains reflect changes in their proteolysis at specific subcellular locations, such as synapses, rather than changes in the overall turnover of these proteins.

A number of studies indicate that the proteolytic products of CAMs are also present at varying levels in the cerebrospinal fluid (CSF) and serum of humans. Western blot analyses with antibodies specific to different portions of these molecules show that these proteolytic products are detectable with the antibodies against the epitopes within their extracellular domains while the intracellular domains are not detectable [60]. These observations indicate that the proteolytic products of CAMs in CSF and serum represent fragments of the extracellular domains of CAMs possibly released to CSF by shedding from the cell surface of neurons in the brain. Proteolytic products of several CAMs have been reported to be increased in CSF and serum of AD patients. For example, CSF levels of L1 analyzed by ELISA have been reported to be significantly increased in AD [60]. This study also reported an increase in the CSF levels of NCAM, which, however, was not statistically significant when compared to normal controls. Increased levels of several proteolytic products of NCAM were also found in the sera of AD patients [61]. In contrast, ELISA analysis has not revealed significant differences in the levels of neuronal cell adhesion molecule (NrCAM), L1 family member, in CSF samples from healthy controls and AD patients [62].

Analysis of the levels of the proteolytic products of CAMs has therefore been proposed to be useful in diagnostics of AD. It should be noted, however, that levels of the proteolytic products of such CAMs as NCAM or L1 in CSF and serum samples of healthy individuals and AD patients overlap considerably [60, 61]. Also, changes in CSF levels of these products are often not specific to AD. For example, levels of the proteolytic products of L1 are also increased in vascular dementia and dementia of mixed type [60]. Levels of the proteolytic products of NCAM are increased in CSF of people suffering from schizophrenia [63] and bipolar mood disorder type I or recurrent unipolar major depression [64], but not in bipolar mood disorder type II patients [64]. However, levels of NCAM are not changed in the serum of patients with autism, although levels of NCAM180 protein but not mRNA are reduced in the brains of these patients [65]. Also, in contrast to AD, CSF levels of L1 are decreased in schizophrenia [63]. Therefore, analysis of specific isoforms and cleavage products derived via different proteolysis pathways might be required to establish proteolytic products of CAMs as markers of specific neurologic conditions including AD.

## 5. Synaptic CAMs as Receptors for A***β*** Oligomers

**Figure 3:**
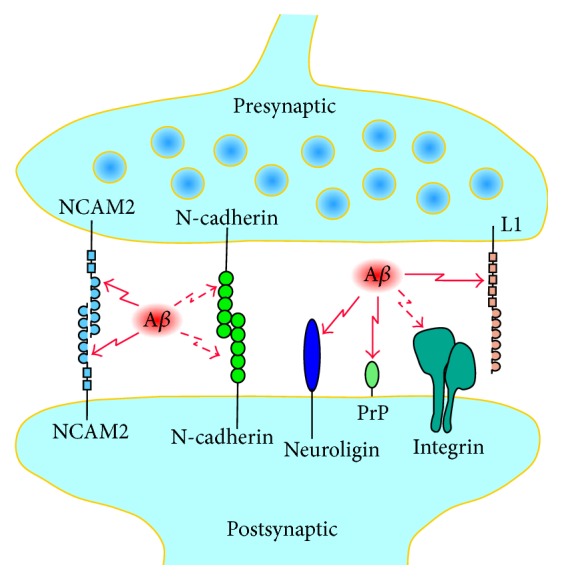
Synaptic CAMs function as receptors for A*β*. Schematic representation of a synapse showing presynaptic and postsynaptic CAMs, which bind to A*β*. Direct interaction with A*β* has been demonstrated for NCAM2, neuroligin, PrP^c^, and L1 (solid red arrows). Binding of A*β* to integrins and N-cadherins is suggested by indirect observations and remains to be confirmed in a direct binding assay (dashed red arrows).

A number of observations indicate that synaptic CAMs act as receptors for A*β* oligomers at the synaptic sites. The extracellular domain of L1 but not the extracellular domain of CHL1 interacts with A*β* in a label-free binding assay [66] (Figure 3). The fibronectin type III homologous repeats 1–3 of the extracellular domain of L1 mediate this effect. Interestingly, the recombinant extracellular domain of L1, but not the recombinant extracellular domain of CHL1, inhibits aggregation of A*β in vitro*. Furthermore, overexpression of L1 by injection of adenoassociated virus encoding L1 decreases the A*β* plaque load, levels of A*β*42, A*β*42/40 ratio, and astrogliosis in a mouse model of AD, which carries human APP with the pathogenic Swedish mutation and the L166P mutated human presenilin-1 [66]. The extracellular domain of NCAM2 also binds to A*β* oligomers both* in vitro* and* in vivo* in cultured mouse hippocampal neurons and in the hippocampus of A*β* generating transgenic mice overexpressing human APP containing the pathogenic Swedish mutation [59].

A*β* oligomers also directly associate with the N-terminus of PrP^c^ both* in vitro* and in the human AD brain, with the binding sites located within residues 23–27 and 95–110 of PrP^c^ [67–71]. Interaction of PrP^c^ with A*β* is a function of A*β* load in the brain and does not depend on PrP^c^ levels [71]. The pathological relevance of this interaction remains, however, to be established. PrP^c^ interacts with other synaptic proteins, including N-methyl-D-aspartic acid- (NMDA-) type glutamate receptors [72] and NCAM [73], and binding of A*β* oligomers to PrP^c^ can interrupt the physiological interactions of PrP^c^ at synapses, resulting in disturbed neuronal communication [74]. PrP^c^ deficient mice are resistant to the neurotoxic effect of A*β* oligomers, and antibodies against PrP^c^ or PrP^c^ peptides prevent A*β* oligomer-induced neurotoxicity indicating that PrP^c^ is involved in the molecular pathways activated by A*β* oligomers to induce neuronal cell death [75]. PrP^c^ traps and concentrates A*β* in an oligomeric form and disassembles mature A*β* fibers [70]. The cleavage fragment of PrP^c^ containing binding sites for A*β* strongly suppresses A*β* toxicity in cultured mouse hippocampal neurons and* in vivo* in mice after intracerebroventricular injections of A*β* [69, 76]. However, memory impairment induced by injection of A*β* oligomers is not reduced in PrP^c^ knockout mice [77], ablation or overexpression of PrP^c^ has no effect on the impairment of hippocampal synaptic plasticity in a transgenic model of AD [78], and synaptic depression, reduction in spine density, or blockade of LTP is induced by A*β* in organotypic hippocampal slice neurons from both wild type and PrP^c^ knockout mice [79]. Therefore, the A*β*-mediated synaptic defects do not require PrP^c^.

Neuroligin-1 is enriched in excitatory synapses and its extracellular domain binds to A*β in vitro* and in cultured rat hippocampal neurons and rat cerebral cortex [80, 81]. A*β* does not interact with neuroligin-2, which is enriched in inhibitory synapses. Neuroligin-1 acts as a nucleating factor during the A*β* aggregation process, stimulating the formation of A*β* oligomers [81]. The soluble extracellular *α*/*β*-hydrolase-fold (ChE-like) domain of neuroligin-1 reduces the A*β*-induced reduction in synaptic density in cultured rat hippocampal neurons and in field excitatory postsynaptic potentials (fEPSP) in rat hippocampal slices possibly by competing with the synaptic neuroligin-1 for binding to A*β* [80].

Indirect observations also suggest that A*β* interacts with integrins since A*β* toxicity was inhibited in human neurons pretreated with adhesion-blocking antibodies against different subunits of integrins, and in particular *β*1, *α*2, and *α*V [82]. Inhibition of *α*1*β*1 integrin has also been shown to reduce A*β* toxicity in rat hippocampal cultures [83]. A*β* toxicity was also inhibited by disintegrin echistatin, a peptide isolated from snake venom that has been shown to inhibit RGD-dependent integrins such as *α*V*β*1 and by integrin ligands such as vitronectin, fibronectin, and superfibronectin suggesting that integrin ligands compete with A*β* for binding to integrins [82]. Application of A*β* also reduces the overall expression of N-cadherin in cultured mouse cortical neurons suggesting that A*β* can bind to N-cadherins [56], although a reduction in N-cadherin proteolysis after application of A*β* has been reported in another study [84].

Interestingly, some CAMs have been shown to interact also with APP. The association of N-cadherin with APP in mouse brains has been shown by coimmunoprecipitation experiments [85]. In an unbiased search for the binding partners of APP using time-controlled transcardiac perfusion cross-linking followed by high stringency immunoaffinity purification and tandem mass spectrometry, several other cell adhesion molecules were identified including PrP^c^ and IgSF CAMs Thy-1, contactin, NCAM1, and neurofascin [86]. In spite of homology to NCAM2, NCAM1 binds to a region of APP which is different to the A*β*-containing region [87] indicating that these interactions may play a role in physiological functions of both molecules. In agreement, contactin-2 has been shown to be a functional ligand of APP. Binding of contactin-2 to APP increases the release of the intracellular domain of APP through *γ*-secretase-dependent cleavage [88]. Contactin-2 competitively inhibits the binding of APP to transforming growth factor *β*2 (TGF*β*2) [89]. Binding of TGF*β*2 to APP induces neuronal cell death [90] and this effect is inhibited by TAG-1 [89] suggesting that TAG-1 regulates interactions of APP with extracellular ligands.

## 6. Synaptic CAMs in Regulation of A***β*** Production

BACE1 is a potential therapeutic target for AD since BACE1 cleavage of APP is the rate limiting step in A*β* production [91]. Synaptic cell adhesion molecules have been shown to play a role in regulation of BACE1 activity. In a high-throughput siRNA screen assessing 15,200 genes for their role in A*β* secretion, LRRTM3 has been identified as a neuronal gene that promotes APP processing by BACE1 [92]. Knockdown of LRRTM3 expression using siRNA results in reduced secretion of A*β* in cultured cells and primary neurons, while overexpression of LRRTM3 increases A*β* secretion [92] suggesting that LRRTM3 promotes BACE1 activity.

In contrast, overexpression of PrP^c^ results in inhibited BACE1-mediated cleavage of APP and reduced A*β* production, while A*β* production is increased in the brains of PrP^c^ knockout mice and in cultured N2a cells after siRNA mediated knockdown of PrP^c^ expression [93] suggesting that PrP^c^ inhibits BACE1 activity. In agreement, in a follow-up study, PrP^c^ has been shown to interact with the prodomain of BACE1 in the trans-Golgi network and regulate targeting of BACE1 to the cell surface and endosomes where it preferentially cleaves APP [94]. PrP^c^ reduces BACE1-mediated cleavage of wild type APP, but not human APP with the Swedish and Indiana familial mutations, suggesting that PrP^c^ may play a role in sporadic AD but not in familial AD [94]. Interestingly, the region at the extreme N-terminus of PrP^c^, which is critical for the interaction or PrP^c^ with BACE-1 and PrP^c^-dependent inhibition of APP-cleaving activity [93], also contains the binding site for A*β* oligomers [67]. These observations suggest that PrP^c^ can play a protective role (inhibition of BACE1) and pathogenic role (binding of toxic A*β* oligomers) in AD and also suggest that the protective function of PrP^c^ can be affected by A*β* oligomers.

An increase in A*β* secretion is also observed in cells cotransfected with N-cadherin [85, 95]. N-cadherin promotes cell surface expression of *γ*-secretase and increases accessibility of *γ*-secretase to APP [95]. Altogether, these observations thus indicate that synaptic CAMs are involved in regulation of the key enzymes involved in A*β* production.

## 7. Effects of Disruptions of Synaptic Adhesion on the Synapse Integrity in AD

Inhibition of N-cadherin function by blocking INP peptides, which mimic a short sequence in the EC1 domain of N-cadherin and thus impair the homophilic transsynaptic interaction of N-cadherin molecules, accelerates the A*β*-induced synapse impairment characterized by a reduction in the frequency of the AMPA receptor-mediated miniature excitatory postsynaptic currents (AMPA mEPSCs) and reduced density of synaptic boutons along dendrites in cultured cortical neurons [57]. Similar effects are observed when N-cadherin function is inhibited by expression of the dominant-negative, truncated N-cadherin lacking the extracellular cadherin domains, or by overexpression of the ectodomain-shed C-terminal fragment of human N-cadherin, which accumulates in AD brains. It is noteworthy that the ectodomain-shed C-terminal fragment of human N-cadherin is further cleaved by *γ*-secretase, and inhibition of *γ*-secretase activity also accelerates the A*β*-induced synapse impairment [57].

Inhibition of N-cadherin function alone has no effect on the numbers of synapses and frequency of AMPA mEPSCs [57]. Interestingly, disruption of NCAM2-mediated synaptic adhesion using recombinant extracellular domains of NCAM2 (NCAM2-ED) results in a reduction in synapse density along dendrites of hippocampal neurons and dispersion of AMPA receptors from synapses [59]. NCAM2-ED accumulates in AD hippocampus and its effect on the synapse integrity is similar to and not additive with the effect of A*β* [59]. It is therefore possible that A*β*-dependent proteolysis of NCAM2 is one of the initial synapse-destabilizing effects of A*β*, which is then followed by disruption of N-cadherin containing adhesion complexes resulting in the complete synapse disassembly.

The complex formed by A*β* and neuroligin-1 also contains GluN2B but not GluN2A subunits of NMDA receptors [80] suggesting that A*β* can directly affect the function of neuroligin-1 in anchoring NMDA receptors at synapses. Whether binding of A*β* to other synaptic CAMs directly contributes to the synapse loss has to be investigated in the future studies.

## 8. Future Directions

While a number of observations indicate that synaptic cell adhesion molecules are affected in AD, our understanding of the molecular and cellular mechanisms underlying these changes and their role in the disease progression is still very incomplete. Further studies assessing levels of synaptic CAMs specifically at synapses are needed to understand whether changes in the overall levels of these CAMs reflect changes in the synaptic adhesion. Whether an increase in the levels of specific proteolytic products of CAMs in CSF and sera of AD patients reflect the A*β*-dependent proteolysis of CAMs at synapses is an interesting possibility which can be analyzed in the future studies. Since synaptic CAMs play key roles in the maintenance of synapse integrity and function by interacting with synaptic scaffolding proteins and neurotransmitter receptors, further analysis of the effects of A*β*-dependent disruption of synaptic adhesion at the synaptic level may help to understand the molecular mechanisms of the initial stages of AD. Furthermore, a number of reports showing that the A*β* toxicity can be reduced by targeting synaptic CAMs indicate that synaptic CAMs deserve further consideration as molecular targets in designing new treatments of AD.
